# Data and Text Mining Help Identify Key Proteins Involved in the Molecular Mechanisms Shared by SARS-CoV-2 and HIV-1

**DOI:** 10.3390/molecules25122944

**Published:** 2020-06-26

**Authors:** Olga Tarasova, Sergey Ivanov, Dmitry A. Filimonov, Vladimir Poroikov

**Affiliations:** 1 Department for Bioinformatics, Institute of Biomedical Chemistry, 107076 Moscow, Russia; smivanov7@gmail.com (S.I.); dmitry.filimonov@ibmc.msk.ru (D.A.F.); vladimir.poroikov@ibmc.msk.ru (V.P.); 2 Department of Bioinformatics of Pirogov Russian National Research Medical University, 107076 Moscow, Russia

**Keywords:** data mining, text mining, virus–host interactions, SARS, SARS-CoV-2, HIV-1

## Abstract

Viruses can be spread from one person to another; therefore, they may cause disorders in many people, sometimes leading to epidemics and even pandemics. New, previously unstudied viruses and some specific mutant or recombinant variants of known viruses constantly appear. An example is a variant of coronaviruses (CoV) causing severe acute respiratory syndrome (SARS), named SARS-CoV-2. Some antiviral drugs, such as remdesivir as well as antiretroviral drugs including darunavir, lopinavir, and ritonavir are suggested to be effective in treating disorders caused by SARS-CoV-2. There are data on the utilization of antiretroviral drugs against SARS-CoV-2. Since there are many studies aimed at the identification of the molecular mechanisms of human immunodeficiency virus type 1 (HIV-1) infection and the development of novel therapeutic approaches against HIV-1, we used HIV-1 for our case study to identify possible molecular pathways shared by SARS-CoV-2 and HIV-1. We applied a text and data mining workflow and identified a list of 46 targets, which can be essential for the development of infections caused by SARS-CoV-2 and HIV-1. We show that SARS-CoV-2 and HIV-1 share some molecular pathways involved in inflammation, immune response, cell cycle regulation.

## 1. Introduction

Studying viruses that infect human beings is vital because viral infections can spread within the global population. For several centuries, measles, poliomyelitis, rotavirus, human immunodeficiency virus, and different respiratory viruses, including influenza, parainfluenza, adenoviruses, and coronaviruses, have attacked humanity. Such viruses as HIV-1 earlier or SARS-CoV-2 nowadays cause the socially significant epidemic or even pandemic diseases [1,2].

SARS-CoV-2 causes severe acute respiratory syndrome and leads to diverse complications, being especially dangerous for people with different comorbidities. Starting from the end of January, COronaVIrus Disease 2019 (COVID-19) has spread all over the world and become a source of fear and sorrow for millions of people. The investigation of the interaction of SARS-CoV-2 with the human body is essential because such information will contribute to the development of effective therapeutic approaches.

According to the study by Gordon [3], the profile of SARS-CoV-2–host interactions is similar to those of involving various viruses, such as West Nile virus, human papillomaviruses, Zika, Dengue, Ebola viruses, and HIV.

The dangerous spreading of SARS-CoV-2 has given rise to multiple attempts of repurposing of drugs initially developed against other viruses, such as hepatitis C, HIV-1, influenza, etc., along with host-targeted drugs such as hydroxychloroquine and azithromycin [4,5,6,7,8,9].

Overcoming the challenges of novel viral infections requires a fast and effective search for novel therapeutic approaches. A perspective strategy is focused on finding proteins involved in the infection process caused by both studied and well-known agents using published articles and other available information. The identified proteins can then be used as pharmaceutical targets. In this study, we aimed at evaluating such approach by considering the extensively explored HIV-1 virus [1] and new dangerous virus SARS-CoV-2 [2].

There are a few experimental and computational approaches examining the possibility of usage of known antiviral drugs, including those employed for the treatment of viral hepatitis C and antiretroviral drugs, to treat SARS-CoV-2 [7,8,9]. There are several studies describing the treatment of SARS-CoV-2 using antiretroviral drugs, such as remdesivir [10], lopinavir/ritonavir, and darunavir [9,11]. The molecular mechanism of action of lopinavir/ritonavir has been discussed [12,13,14]. Their application for the treatment of COVID-19 as monotherapies [12] or in combination with other drugs [13] has been shown.

While the mechanisms of action of antiretroviral drugs against COVID-19 are under investigation, there are data suggesting that SARS-CoV-2 may affect the immune system [15]. Lymphopenia can be present in patients with COVID-19 [16] and can be associated with disease progression [16].

The analysis of the available information provides (1) data on repurposing of antiretroviral drugs against SARS-CoV-2, (2) data showing similarity of interaction profiles between SARS-CoV-2 and human and HIV-1-human, (3) data indicating common pathophysiological mechanisms between SARS-CoV-2 and HIV-1 infections. In addition, there are a lot of data on the molecular mechanisms of HIV infection, regarding multiple pathways of virus–host interactions and the development of novel therapeutic approaches. Text and data mining approaches can be helpful for fast and accurately extracting information about chemical compounds and their biological activities, as well as proteins associated with molecular mechanisms of disease development [17,18]. In this study, we applied text and data mining approaches to identify possible molecular pathways shared by HIV-1 and SARS-CoV-2. Our hypothesis was that the search for similar molecular mechanisms shared by SARS-CoV-2 and HIV-1 could shed light on novel potential therapeutic approaches for the treatment of COVID-19.

SARS-CoV-2 and HIV-1 belong to different virus classes, according to Baltimore classification [19]. Data on the homology between SARS-CoV-2 and HIV-1 are controversial [20]. Besides, they use different cells to replicate in the human body. HIV-1 uses the receptors CCR5 or CXCR4 to infect CD4 T cells [21,22], while SARS-CoV-2 uses ACE2 to infect the cells of heart, lungs, kidneys, and gastrointestinal tract [23].

There are therapeutic strategies to combat HIV-1 infection in addition to combination antiretroviral therapy, which is currently used to treat HIV-infection [24]. Other approaches to combat HIV-1 infection include novel strategies such as disrupting the *CCR5* gene in human hematopoietic stem/progenitor cells (HSCs) [25] using zinc finger nuclease-mediated CCR5 knockout, or in CD4^+^ T cells or HSCs using CRISPR/Cas9 genome editing [26,27]. Viral RNA genome degradation and inhibition of viral gene expression is another promising strategy to prevent the replication of SARS-CoV-2 [28] and HIV-1 [29]. The search for potent low-molecular-weight drugs targeting SARS-CoV-2 is ongoing, but some currently applied treatment strategies include the use of some known drugs such as remdesivir, chloroquine, umifenovir, and some antiretroviral drugs (darunavir, lopinavir in combination with ritonavir) [30].

The therapeutic effects of medicines applied for the treatment of rheumatoid arthritis [31] along with those of some other drugs such as antiretroviral drugs (darunavir, lopinavir in combination with ritonavir) [32] on SARS-CoV-2 have generated interest in identifying similar mechanisms of viral infection shared by SARS-CoV-2 and HIV-1, in particular their effects on the host’s organism. Another important fact is that SARS-CoV-2 can cause serious complications in people with comorbidities [11,33,34,35], in particular, by causing the so-called cytokine release syndrome [33].

In this study, we will also discuss the role of the identified virus–host interactions in the pathogenesis of SARS-CoV-2 and the molecular mechanisms shared by HIV-1 and SARS-CoV-2.

## 2. Results

We collected a set of proteins, identified in previous papers, relevant to both HIV–host and SARS-CoV-2–host interaction. We searched them using different protein and gene databases, including UniProt, KEGG, DisGeNet, and Integrity [36,37,38,39]. Some examples of the most significant proteins that play a role in both HIV-1 and SARS-CoV-2 infections are presented in Table 1.

For a full list, see Supplementary Materials, Table S1.

The possible role of the identified proteins in the molecular mechanisms of these infections was analyzed.

**Figure 1:**
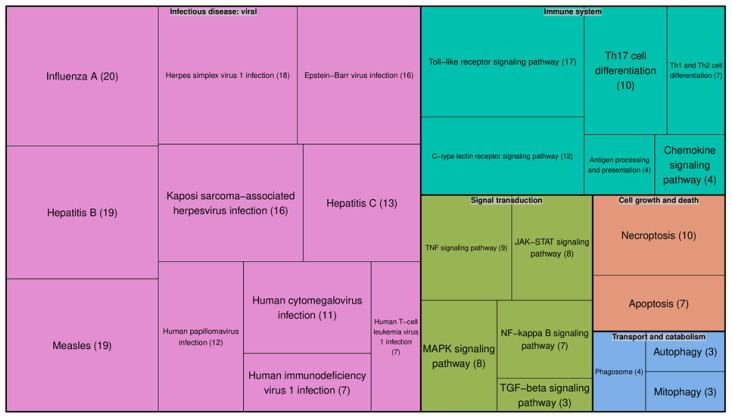
KEGG pathways enriched in the genes associated with the human proteins found by our analysis. Each color represents an individual pathway. The size of each box reflects the number of proteins involved in that particular pathway. The number of proteins involved in each pathway is given in brackets.

Besides the interpretation of the role of individual proteins in SARS-CoV-2 and HIV-1 infections, we performed KEGG [37] pathway enrichment analysis using genes related to the human proteins found in UniProt: we obtained 46 proteins (see Materials and Methods). Then, we manually inspected the positions of the genes in the maps of enriched pathways and filtered out non-relevant pathways. For example, the FoxO signaling pathway was identified by the enrichment analysis, but FOXO transcriptional factors were not among those 46 proteins; thus, we considered the pathway as non-relevant. Finally, we obtained a list of 27 pathways (Figure 1):

The significant number of signaling pathways related to the immune system and the Toll-like receptor signaling pathway included the largest number of proteins identified. There were several pathways related to various viruses. This means that some of the 46 proteins are involved in pathways shared by SARS-CoV-2, HIV-1, and other viruses. This result was confirmed by a disease enrichment analysis, which was performed based on gene–disease associations using the DisGeNet database [38]. Various viral infections and related diseases, including SARS-CoV-2 and HIV-1 infections, were among the top enriched diseases (see Supplementary Materials, Figure S1).

**Figure 2:**
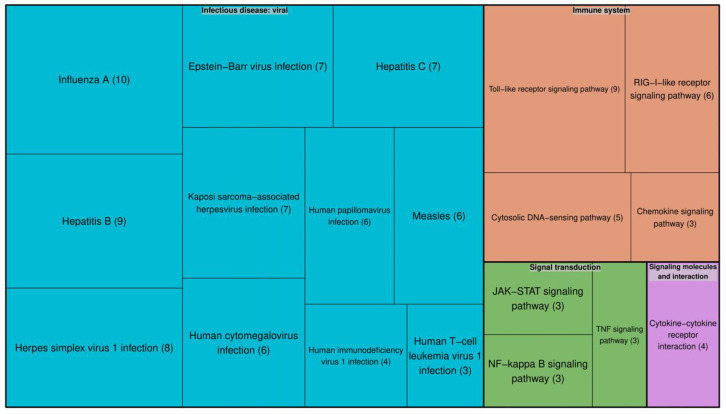
KEGG pathways enriched in the genes associated with human proteins involved in both SARS-CoV-2–host and Dengue–host interactions. Each color represents an individual pathway. The size of each box reflects the number of proteins involved in that particular pathway. The number of proteins involved in each pathway is given in brackets.

To check whether other viruses have similar interactomic profiles to that of SARS-CoV-2, we also evaluated texts associated with Dengue virus and determined an overlap between the proteins relevant to Dengue–host interactions and those relevant to SARS–CoV-2–host interactions. We found 32 human proteins, which can be involved in both SARS-CoV-2–host and Dengue–host interactions. For them, we prepared a list of related pathways. We observed a similar profile of molecular pathways for proteins shared by Dengue virus and SARS-CoV-2 to that for proteins shared by HIV-1 and SARS-CoV-2 (Figure 2).

Nevertheless, we found differences in the number of pathways related to the immune system, in particular in the number of proteins associated with the Toll-like receptors-related pathway; this is consistent with other studies showing the involvement of the immune system in SARS-CoV-2 disease progression [15,16]. Also, some of the pathways, for instance, necrosis, apoptosis, and autophagy pathways, common to SARS-CoV-2–host and HIV-1–host interactions were not found in the list of pathways related to SARS-CoV-2–host and Dengue–host interactions. This information on proteins involved in the interactions between various viruses and the human organism can be helpful for the future development of therapeutic strategies against these viruses.

In addition, by examining the 46 proteins related to both SARS-CoV-2-host and HIV-1-host interactions in the Integrity database, we identified the main processes associated with targets currently validated for the treatment of different pathological conditions and diseases. We discuss our results below.

## 3. Discussion

By a literature review and a pathway enrichment analysis, we identified some key proteins and pathways which may be proposed as essential for both SARS-CoV-2 and HIV-1 infections. For instance, the protein AIP4 may play a role in inflammation, and the interaction of SARS-CoV-2 with this protein may downregulate the host’s interferon response [40]. This would lead to the disruption of the host’s immune response. A similar mechanism was reported for HIV-1 [41].

It has been shown that protein beclin 1 induces autophagy in immune cells, which negatively regulates the host’s immune defense mechanisms. The possible role of beclin-1 in autophagy as one of the molecular mechanisms of viral pathogenesis was discussed for both coronaviruses and HIV-1 [42,43]. The inhibition of beclin-1 can help to reduce viral replication of MERS-CoV (Middle East respiratory syndrome coronavirus) [44]. Despite these findings, it is supposed that the viruses can either escape autophagy or use the autophagic machinery for their own replication [45]. That is why the strategy of inhibition of the proteins included in the autophagic machinery is not considered as a promising one to combat viral infections including HIV-1 infection and COVID-19 [45].

The proteins cathepsin B and L, which are endosomal proteases, are responsible for viral fusion and the release of the viral genome into the cytoplasm of human cells [46]. The dependence of SARS-CoV-2 entry on cathepsin B was shown in the study by Hoffman et al. [47] and discussed in also elsewhere [47,48]. This process was shown to be pH-dependent. Divani et al. [48] suggested that the Spike (S) protein of the SARS-CoV-2 can be “primed” by cathepsins B and L, which is essential for their penetration into the cells. In the study by Yoshii. et al. [49], the authors claimed that cathepsin B is involved in CD4-independent entry of HIV-1, i.e., this type of entry can be used by HIV-1 variants that do not require CD4 for infection [49]. There is evidence that an interaction of SARS-CoV-2 with cathepsin L is essential for SARS-CoV-2 entry [50]. Selective inhibitors of cathepsin L can be considered for the therapy of COVID-19 [51]. The authors suggest the usage of selective cathepsin L inhibitors because active cathepsin S can assist viral release and MHC-I- and MHC-II-mediated antigen presentation as well as T-cell activation, which is important for protective immunity against SARS-CoV-2 and other infections. It is interesting that for herpesviruses, the role of cathepsin L in releasing viral particles was reported [52], giving rise to the hypothesis that therapeutic strategies directed to inhibiting pathways associated with cathepsin L can be beneficial for non-specific antiviral activity. Cathepsin B is also associated with the release of HIV-1 particles in blood monocyte-derived macrophages [53]. Additionally, it was shown that secretion of cathepsin B by HIV-infected macrophages leads to neuronal internalization and induces the progression of neuronal dysfunctions [54,55]. Therefore, cathepsin B expressed in different tissues is involved in several different molecular pathways of disease progression.

E3 ubiquitin ligase TRIM25 participates in the regulation of the antiviral innate immunity [56,57]. In particular, the SARS-CoV nucleocapsid protein, which has over 90% identity to that of SARS-CoV-2 [58] was found to bind TRIM25, thereby inhibiting type 1 interferon production [56]. TRIM25 was also shown to be required for the activity of a type I interferon-inducible host factor ZAP, which specifically inhibits the replication of certain viruses, including HIV-1, Sindbis virus, and Ebola virus [57].

Some proteins, such as interferon gamma, interferon regulator factor 3, interleukin 2, matrix metalloproteinases, are responsible for the immune response or inflammation [59,60]. Therefore, these proteins should be involved in specific or non-specific antiviral or inflammatory response. An increase of interleukins in the blood plasma occurs in response to the infection, especially it can be observed during viral infections. SARS-CoV-2 affects the immune response, leading to a significant increase of chemokines and cytokines. To combat the cytokine storm in patients with COVID-19, several anti-rheumatic medicines, chloroquine, hydroxychloroquine, as well as JAK inhibitors, IL-6 inhibitors, IL-1 inhibitors, anti-TNF-α agents, and corticosteroids are considered [59]. The interleukin-6 receptor antagonist Tocilizumab is described as one of the promising drugs to combat the cytokine storm [59]. The development of the cytokine storm in the patients infected by HIV-1 was shown [61].

Interestingly, the role of the c3-complement system activation in SARS-CoV-1, SARS-CoV-2-related disorders and HIV-related comorbidities was discussed [62,63,64,65]. According to our results, complement c3 is involved in both inflammation and immune response processes, besides its role as a complement activation. An attempt to combat COVID-19 using the compstatin-based complement c3 inhibitor AMY-101 has been shown recently [65], resulting in a good clinical response. The involvement of c3 complement in the mannose binding lectin-pathway (MBL) was shown during membrane attack complex (MAC) formation, leading to cell lysis and death [66] of HIV-infected patients.

The genes encoding the proteins presented in Table 1 are expressed widely or ubiquitously. Therefore, they can participate in similar pathways during both SARS-CoV-2 and HIV-1 infections, despite the fact that SARS-CoV-2 and HIV-1 use different cell types for their replication, which indicates different molecular basis for the progression of their associated diseases. Most of the proteins identified are *Homo sapiens* proteins. For them, similar or identical molecular mechanisms of pathological processes during HIV-1 and COVID-19 can be proposed. This observation confirms the reliability of our approach for the extraction of data on proteins involved in the interaction of the host with either SARS-CoV-2 or HIV-1.

When we manually checked about 100 named entities (NE), we found 10 NE; and the lexical meaning of them was not related to known proteins but it was associated with the biological object, such as a virus, an organism, etc. Therefore, about 90% of the proteins found using our text and data mining algorithm can be considered as potential targets involved in the pathogenesis of both SARS-CoV-2 and HIV-1 infections.

**Figure 3:**
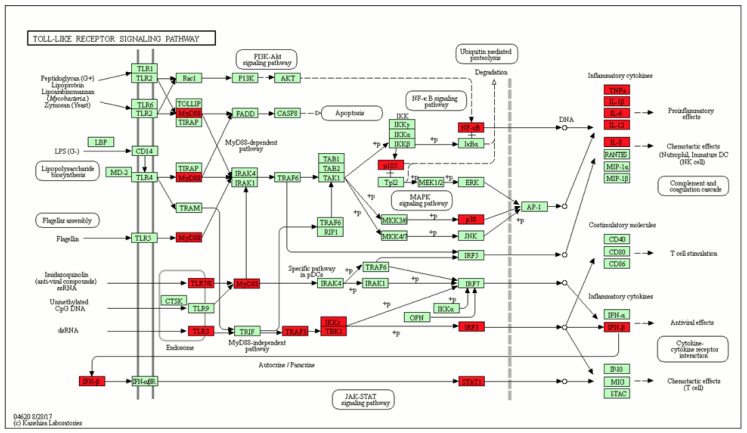
The Toll-like receptor signaling pathway includes the highest number of the proteins identified, related to immune system pathways and appearing in both SARS-CoV-2 and HIV-1 infections.

Pathway enrichment analysis can help to identify a role of some signaling pathways in both HIV-1 ad SARS-CoV-2 infection. To perform the pathway enrichment analysis, first we manually mapped the initial entities, extracted by text mining, to UniProt Accession numbers. This process allowed us to collect a list of 46 human proteins. The Toll-like receptor-signaling pathway has the highest number of proteins related to immune system pathways and found to appear in both SARS-CoV-2 and HIV-1 infections (Figure 3). 

Toll-like receptors 3 (TLR3) and 7 (TLR7) play an important role in the innate immune response against HIV-1 and SARS-CoV-2 [67,68,69,70,71]. The polymorphism rs3775291 of the TLR3 receptor is associated with protection against HIV-1 [67,68]. TLR3 agonists increase the anti-HIV immune response induced by vaccination [69]. TLR7 agonists increase the anti-HIV-1 immune response, the activation of HIV-1 expression, and the inhibition of HIV-1 replication, which may help to treat latent infections [70]. The role of TLR3 and TLR7 in the pathogenesis of COVID-19 is discussed in the study by Sallenave [71]. 

**Figure 4:**
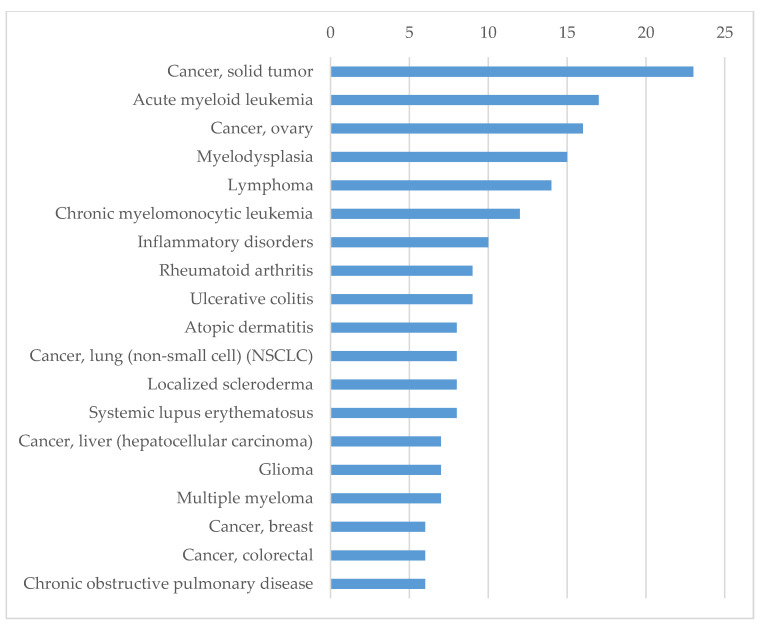
The distribution of diseases and pathological conditions by the number of protein targets associated with them.

Based on 46 human proteins, for which records were found in KEGG, we identified the biological processes related to diseases and the pathological conditions associated with these proteins using the Integrity database. We analyzed the sets of currently validated targets for the treatment of certain diseases and pathological conditions. According to these results, the majority of proteins are related to inflammation, autoimmune disorders and cancer (Figure 4).

It seems surprising that most of the found targets of existing drugs are not related to infectious diseases but, rather, are associated with inflammation, cancer, or immune disorders. However, since we analyzed virus–host interactions, we could expect that SARS-CoV-2 and HIV-1 interaction with the host would be associated with processes like inflammation and those that affect the host immune system. The association between cancer development and inflammation has been discussed in several studies [72,73,74,75]. Moreover, the role of the targets associated with autoimmune diseases in the development of cancer has been described [74]. Based on the analysis of the targets strongly related to the found conditions according to the Clarivate Analytics Integrity database [39]—provided in the Supplementary Materials (Table S2)—we identified three main groups of proteins. The first group contains interleukins (IL), interferon (IFN), interferon regulatory factor (IRF), IL6, IL8, IL12a, IL12b, IL18, IRF3. For these proteins, their role in inflammation and the immune response during infection is well studied [76]. The second group contains a few proteins, such as the Toll-like receptors (TLR) TLR3 and TLR7, whose role in the immune response is described above. The third group contains some transcription factors and kinases which play a role in various molecular processes that are related to the inflammation response. They also can be important for the development of cancer. Regarding pathological conditions, in general, the results found using Integrity are very similar to those found using UniProt and KEGG. Still, an interpretation of the results collected from Integrity can help to better understand the effectiveness of approved drugs for the treatment of SARS-Co-V2 when specific antiviral medicines are not known.

The obtained results were confirmed by disease enrichment analysis based on gene–disease associations using the DisGeNet database. We manually analyzed the top 30 enriched disease terms and classified them in four groups (see Supplementary Materials, Figure S1). The first group includes 12 terms related to viruses and bacteria, e.g., SARS, HIV infections, hepatitis B and C, cytomegalovirus infections, influenza, tuberculosis, and others. The second group is related to inflammatory respiratory diseases: asthma, chronic obstructive airway disease, and pneumonia. The third group is related to cancer diseases, whereas the fourth group includes all other diseases, which are associated with inflammation, e.g., atherosclerosis, systemic lupus erythematosus, and rheumatoid arthritis. All 46 proteins appeared to be associated with at least one of the selected diseases, and the cytokines CXCL8, IL6, TNF were associated with all of them.

Additionally, we provide the full list of proteins related to the development of SARS-CoV-1 infection only in Table S3 of the Supplementary Materials.

Summarizing our findings based on the analysis of the literature and on data retrieved from biological databases, we can define groups of proteins, which can be essential for both SARS-CoV-2-host and HIV-1-host interactions. The first group represents proteins responsible for viral entry in human cells, which are the same for HIV-1 and SARS-CoV-2 as well as for some other viruses. The second group of proteins is associated with the host’s immune response to virus invasion of the cells; HIV-1 or SARS-CoV-2 interaction with these host proteins will lead to immune suppression. In addition, there are a few proteins that were found in just one or two publications but are important for processes such as blood coagulation. Third, some viral proteins can interact with human proteins in a close manner or share some similar structural motifs and can have the same effect on protein–host interaction. 

## 4. Materials and Methods

**Figure 5:**
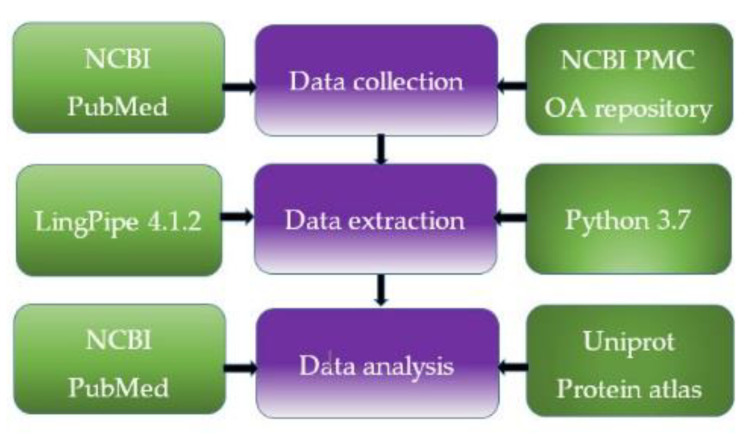
General scheme of the data extraction and analysis.

We applied text and data mining algorithms to extract from publications a list of proteins relevant to virus–host interactions common for HIV-1 and SARS-CoV-2. For this purpose, we performed several stages of text processing and data analysis (Figure 5).

In the first stage, we collected abstracts and full texts from NCBI PubMed and NCBI PubMed Central (PMC) repositories. We used the application-programming interface (API) available for PubMed and PMC, based on query allowing automated filtering of relevant papers. PubMed contains over 27 million articles, and PMC includes several million articles; for some of them (the so-called Open Access (OA) subset), it is possible to retrieve full texts in a machine-readable format (Extensible Markup Language, XML). We used PubMed and the Open Access Subset of PMC for our computational experiments. Relevant documents were selected based on the following combination of keywords: “HIV-1 AND virus–host interactions”, “SARS AND virus–host interactions,” “SARS-CoV-2 AND virus–host interactions”. The queries were built using Python 3.7 scripts. We obtained the list of the identifiers of articles in PubMed electronic library (PMIDs) or the identifiers of NCBI PubMed Central (PMCIDs). The queries allowed identifying over 200 PMIDs and over 6000 PMIDs in the PubMed database relevant to SARS–host and SARS-CoV-2-host interactions and HIV1–host interactions, respectively. We named these corpora of texts as “SARS-PubMed” and “HIV-PubMed”. Over 180 PMCIDs were retrieved for SARS–host and SARS-CoV-2–host interactions, and over 57,000 PMCIDs were collected for HIV-1–host interactions using an automated query (the “SARS-PMC” and “HIV-PMC” corpora). We excluded reviews from further consideration. Using PubMed API, we collected the texts of over 6200 abstracts of PubMed and over 38,000 full texts of original articles in XML.

We used the automatically collected abstracts for the corpora “SARS-PubMed” and “HIV-PubMed” and the full texts collected for the corpora “SARS-PMC” and “HIV-PMC”. The texts represented in XML format were processed to save only the Materials and Methods, Results or Results and Discussion sections; the Introduction section was excluded. We used the abstracts of the “SARS-HIV-PubMed” corpus in the current study because the license agreement restricts the availability of full texts. It does not lead to a collection of unbiased corpora (i.e., corpora which are not limited by using several journals). Full texts of SARS-PMC and HIV-PMC were used for data extraction.

The collected corpora of abstracts were processed using Lingpipe-4.1.2 named entity algorithm trained on GENIA corpus [77]. For evaluating the average accuracy of recognition, we applied five-fold cross-validation based on the corpus used for training the algorithm and an external test set “HIV-RT-inhibitor corpus” containing 250 abstracts. The average precision for Lingpipe-4.1.2 was 0.84%, the recall was 0.79.

After the extraction of the sets of proteins using all the corpora, we automatically compared them and identified overlapping proteins. According to our hypothesis, the proteins found in both sets of texts that were relevant (a) to HIV-1–host interactions and (b) to SARS-host and SARS-CoV-2–host interactions can influence the pathways and molecular mechanisms responsible for infection, invasion, and disease progression similarly or identically for HIV-1 and SARS-CoV-2.

The final set of proteins was checked using the UniProt API [78,79]. Only proteins found in UniProt and associated with *Homo Sapiens*, SARS-CoV-2, HIV-1 were saved in the list of proteins, which we analyzed further. The manual inspection of the proteins selected was carried out using NCBI PubMed electronic library and the UniProt, Protein Atlas, and Integrity databases.

To identify signaling pathways, first we manually mapped the initial entities, which were extracted by text-mining, to UniProt Accession numbers and obtained a list of 46 human proteins. Pathways enriched with 46 genes, were identified from the KEGG database [18] using the “Enrichr” R package. We selected pathways which included at least 3 genes from the 46 ones and adjusted the *p*-value to less than 0.05. Visualization of the list of pathways was performed using the “Treemap” R package. Additionally, we identified diseases associated with 46 genes using Enrichr and gene–disease associations using the DisGeNet database (https://www.disgenet.org) [38].

## 5. Conclusions

We demonstrated the usefulness of text and data mining techniques for finding human proteins involved in similar pathological processes involving well-known and novel viruses. We used the text and data mining techniques to identify possible molecular pathways common to two very distinct viruses, HIV-1 and SARS-CoV-2, as a case study. We extracted data for proteins related to the molecular mechanisms that may be essential for the development of the infections caused by SARS-CoV-2 and HIV-1 and demonstrated the usefulness of such application. We found a few key proteins that are known to be associated with mechanisms related to both HIV-1 and SARS-CoV-2 infections and to several different processes of pathogenesis, from virus entry to evasion of immune response and regulation of inflammation in the host. We considered the particular properties and molecular pathways that can be involved in different steps of HIV-1 and SARS-CoV-2 infections. We provided a few examples of therapies targeting some of the identified proteins. We showed that even these unrelated viruses can share the same human proteins and regulatory pathways, which should be taken into account for the development of treatment strategies based on existing drugs. We believe that the suggested approach can be used to select known drugs to be applied against novel viruses on the basis of shared regulatory pathways and protein targets.

## Figures and Tables

**Table 1:** Examples of proteins that can have a role in both HIV-1 and severe acute respiratory syndrome (SARS)-coronavirus (CoV-2) infections.

Protein Name	UniProt ID ^1^	Species ^2^	Tissue ^3^	Process
AIP4	Q96J02	*Homo sapiens*	Widely expressed	Inflammation
Beclin 1	Q14457	*Homo sapiens ^1^*	Ubiquitous	Autophagy of immune cells
Cathepsin B	P07858	*Homo sapiens*	Widely expressed	Entry of the virus Viral replication (HIV-1)
Cathepsin L	Q5K630	*Homo sapiens*	Widely expressed	Entry of the virus
Complement C3	P01024	*Homo sapiens*	Blood plasma and over 200 tissues	Immune response Inflammation Complement activation
IFITM1	P13164	*Homo sapiens*	Bone and over 200 tissues	Immune response

^1,2,3^ UniProt ID, species, tissue are the identifiers of proteins in UniProt database.

## Supplementary Materials

The following are available online, Figure S1: Four groups of diseases formed on the basis on gene-disease associations from DisGeNet database, Table S1: The list of human proteins identified as having impact on SARS-CoV-2 and HIV-1 infections development, Table S2: The proteins found as belonging to the set of validated targets for treatment pathological conditions and diseases according to the Integrity database, Table S3: List of human proteins involved in the development of the infection caused by SARS-CoV-1 according to the text and data mining.
