# Supplementary material for: Data and Text Mining Help Identify Key Proteins Involved in the Molecular Mechanisms Shared by SARS-CoV-2 and HIV-1
Source: Molecules. 2020 Jun 26;25(12):2944. doi: 10.3390/molecules25122944 (PMC7357070; doi:10.3390/molecules25122944)
Supplement: Supplementary file 1 [file molecules-25-02944-s001.pdf]

Supplementary Materials to the communication "Data and text mining help identify key proteins involved in the molecular mechanisms shared by SARS and HIV"

Table S1. The list of human proteins identified as having impact on SARS-CoV-2 and HIV-1 infections development.

UniProtAC	Gene symbol	Protein name	EntrezID	Terms	Reference PubMedID/ PMCID*
Q9BYF1	ACE2	Angiotensin-converting enzyme 2	59272	ACE2; ACE2) protein; angiotensin converting enzyme 2; Angiotensin-converting enzyme 2	32469255
P01024	C3	Complement C3	718	C3	PMC7214348 32359943
Q9H2X3	CLEC4M	C-type lectin domain family 4 member M	10332	L-SIGN, CD299	32417709 32318328
P07858	CTSB	Cathepsin B	1508	cathepsin B	PMC7214348
P07711	CTSL	Cathepsin L1	1514	cathepsin L	PMC7214348
P10145	CXCL8	Interleukin-8	3576	IL-8	PMC7255975
P19525	EIF2AK2	Interferon-induced, double-stranded RNA-activated protein kinase	5610	PKR	32360182
Q14152	EIF3A	Eukaryotic translation initiation factor 3 subunit A	8661	eIF3	32442437
P15311	EZR	Ezrin	7430	ezrin	32397911
P11021	HSPA5	Endoplasmic reticulum chaperone BiP	3309	GRP78	32340551
P04792	HSPB1	Heat shock protein beta-1	3315	HSP27	32398026 32268515
P13164	IFITM1	Interferon-induced transmembrane protein 1	8519	IFITM1	32376402 32413319
Q01628	IFITM3	Interferon-induced transmembrane protein 3	10410	IFITM3	PMC7255975
P01574	IFNB1	Interferon beta	3456	interferon-β	32152082 32275178
Q14164	IKBKE	Inhibitor of nuclear factor kappa-B kinase subunit epsilon	9641	IKKε	32179480
P29459	IL12A	Interleukin-12 subunit alpha	3592	IL-12	32335367
P29460	IL12B	Interleukin-12 subunit beta	3593	IL-12	32335367
Q14116	IL18	Interleukin-18	3606	IL-18	32376392

P01584	IL1B	Interleukin-1 beta	3553	IL-1β	PMC7255975
P60568	IL2	Interleukin-2	3558	interleukin 2; interleukin-2	PMC7255975
P05231	IL6	Interleukin-6	3569	IL-6	PMC7255975 PMC7237916
P10914	IRF1	Interferon regulatory factor 1	3659	IRF-1	32450346
Q14653	IRF3	Interferon regulatory factor 3	3661	IFN regulatory factor 3	32169119 32446778
Q96J02	ITCH	E3 ubiquitin-protein ligase Itchy homolog	83737	AIP4	32442437
Q9H492	MAP1LC3 A	Microtubule-associated proteins 1A/1B light chain 3A	84557	LC3-I (light chain protein 3-I	32285908
Q16539	MAPK14	Mitogen-activated protein kinase 14	1432	p38 MAPK	32376402
Q7Z434	MAVS	Mitochondrial antiviral-signaling protein	57506	MAVS	PMC7225364 32353634
P11226	MBL2	Mannose-binding protein C	4153	MBL	32461141 32467443
Q99836	MYD88	Myeloid differentiation primary response protein MyD88	4615	MyD88	32467561
P19838	NFKB1	Nuclear factor NF-kappa-B p105 subunit	4790	NF-κB	32442437
P09874	PARP1	Poly [ADP-ribose] polymerase 1	142	PARP	32317220
P62937	PPIA	Peptidyl-prolyl cis-trans isomerase A	5478	cyclophilin A	PMC7237916
P84022	SMAD3	Mothers against decapentaplegic homolog 3	4088	SMAD3	32194980 32405226
Q14457	BECN1	Beclin-1	8678	Beclin1	PMC7231166 PMC7199282 32285908
P42224	STAT1	Signal transducer and activator of transcription 1-alpha/beta	6772	STAT1	32346093
P40763	STAT3	Signal transducer and activator of transcription 3	6774	STAT3	32413319
P42226	STAT6	Signal transducer and activator of transcription 6	6778	STAT6	PMC7239618
Q9UHD2	TBK1	Serine/threonine-protein kinase TBK1	29110	TBK1	32346093
P01137	TGFB1	Transforming growth factor beta-1 proprotein	7040	TGF-β1	32470547

O15455	TLR3	Toll-like receptor 3	7098	TLR3	PMC7242964
Q9NYK1	TLR7	Toll-like receptor 7	51284	TLR7	PMC7242964
O15393	TMPRSS2	Transmembrane protease serine 2	7113	serine 2	32165541<br>32470547<br>32302590
Q14258	TRIM25	E3 ubiquitin/ISG15 ligase TRIM25	7706	EFP	28148787<br>32416961
P01375	TNF	Tumor necrosis factor	7124	TNF	32346093
P04637	TP53	Cellular tumor antigen p53	7157	p53	32376392<br>32228226
Q13114	TRAF3	TNF receptor-associated factor 3	7187	TRAF3	32276453
P08670	VIM	Vimentin	7431	vimentin	32405226<br>32172672

* The NCBI PubMed or NCBI PMC identifier of the publication, which confirms the host-interactions between SARS-CoV-2 and human.

Table S2. The proteins found as belonging to the set of validated targets for treatment pathological conditions and diseases according to the Integrity database.

N	Function
1	Interleukin-6 (IL-6) is a member of a subfamily of related hematopoietic cytokines that include leukemia inhibitory factor, ciliary neurotrophic factor, oncostatin M, cardiotrophin-1 and IL-11.
2	TNF-alpha is a proinflammatory cytokine (also known as cachectin) and is a member of the TNF family of cytokines that is released by activated macrophages and lymphocytes.
3	p53 is a transcription factor that modulates stress responses and which, upon activativation, precedes apoptosis in many cell types.
4	STAT3 (also known as Acute-phase response factor) is a member of the STAT (Signal Transducers and Activators of Transcription) family of transcription factors that binds to IL-6-responsive elements present on the promoters of various genes involved in acute-phase responses.
5	PARP-1 is nuclear enzyme that signals the presence of DNA damage by catalyzing the addition of ADP-ribose units to DNA, histones, and various DNA repair enzymes and by facilitating DNA repair.
6	IL-1beta
7	p38 alpha MAPK (MAPK14), p38 beta MAPK (MAPK11), p38 gamma MAPK (MAPK12) and p38 delta MAPK (MAPK13 or SAPK4) which are activated by a variety of cellular stresses including osmotic shock, inflammatory cytokines, lipopolysaccharides (LPS), ultraviolet light and growth factors.
8	TLR7 is a member of the toll-like receptor (TLR) family involved in pathogen recognition and innate immunity activation.
9	IL-8 is an ELR+ (Glu-Leu-Arg) CXC chemokine suggested to be an important mediator of angiogenesis that may contribute to
10	STAT-6 is a member of the STAT family of transcription factors. It is activated (phosphorylated) by IL-4 after which it migrates to the nucleus where it activates the transcription of target genes.
11	IL-2 is a cytokine produced by CD4+ T lymphocytes upon activation by antigens and costimulators.
12	TLR3 is a member of the toll-like (TLR) receptor family which includes key recognition structures of the innate immune system.

13	IL-18 is a member of the IL-1 family. It enhances the activity of natural killer (NK) cells and plays a major role in granulocyte/macrophage colony-stimulating factor (GM-CSF) production, T cell proliferation and Th1 cytokine production.
14	IL-12A is the apha subunit of the IL-2 complex, a cytokine that signals through its receptor via the JAK/STAT pathway and may have a role in some autoimmune diseases.
15	IL-12B is the beta subunit of the IL-2 complex, a cytokine that signals through its receptor via the JAK/STAT pathway and may have a role in some autoimmune diseases.
16	C3 is a central component of the complement cascade in both classical and alternative pathways.
17	PPIase A is an enzyme that catalyzes the cis-trans isomerization of proline imidic peptide bonds in oligopeptides.
18	TMPRSS2 is a serine protease that proteolytically cleaves and activates the viral spike glycoproteins which facilitate virus-cell membrane fusions.
19	TGF-beta1 is a cytokine that regulates cell proliferation, differentiation and apoptosis.
20	IKK-E phosphorylates IkappaB which causes its dissocation from the IkappaB/NF-kappaB complex; itbis then degraded. It is invovled in the immune response.
21	Cathepsin B is a lysosomal acid hydrolase that is involved in the proteolytic processing of amyloid precursor protein (APP).
22	ACE2 is a carboxypeptidase which converts angiotensin I to angiotensin 1-9, a peptide of unknown function, and angiotensin II to angiotensin 1-7, a vasodilator..
23	Vimentin is a class-III intermediate filament found in various nonepithelial cells, especially mesenchymal cells.

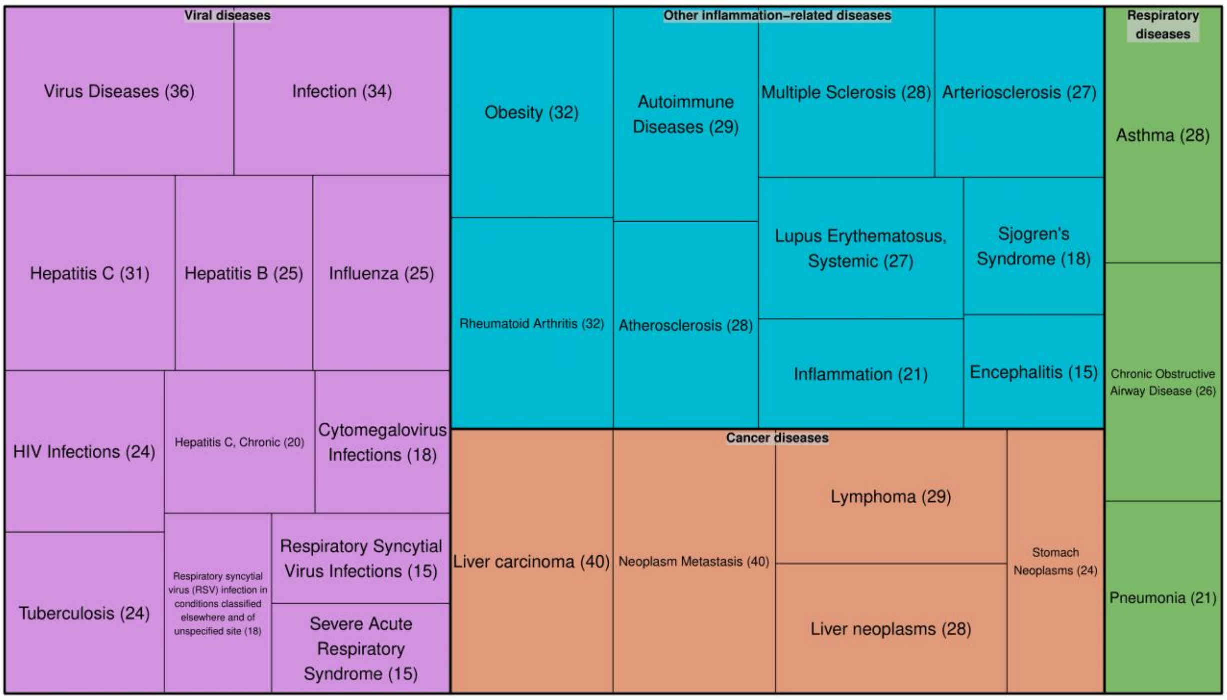

Figure S1. Four groups of diseases formed on the basis on gene-disease associations from DisGeNet database.

Table S3. List of human proteins involved in the development of the infection caused by SARS-CoV-1 according to the text and data mining.

Name found	Uniprot_ID	Protein Name	Gene Name
ACE2	Q9BYF1	angiotensin converting enzyme 2	ACE2 UNQ868/PRO1885
Allergin-1	Q7Z6M3	Allergin-1 (Allergy inhibitory receptor 1)	MILR1
CAMK2D	Q13557	Calcium/calmodulin-dependent protein kinase type II	CAMK2D
C3	P01024	Complement C3	C3, CPAMD1
cathepsin B	P07858	Cathepsin B	CTSB
cathepsin L	P07154	Cathepsin L	Ctsl
CD299	Q9H2X3	C-type lectin domain family 4 member M	CLEC4M
co-chaperone Cdc37	Q16543	Hsp90 co-chaperone Cdc37	CDC37
Serglycin	P10124	Serglycin	SRGN
CP47	Q13515	Phakinin	BFSP2
DC-SIGN	Q9NNX6	CD209 antigen	CD209, CLEC4L
cyclophilin A	P62937	Peptidyl-prolyl cis-trans isomerase A, (Cyclophilin A)	PPIA
RyDEN	Q9NUL5	Shiftless antiviral inhibitor of ribosomal frameshifting protein	SHFL
EGFR	P00533	Epidermal growth factor receptor	EGFR
E3 ubiquitin ligase	P62877	E3 ubiquitin-protein ligase RBX1	RBX1
ezrin	P15311	Ezrin (Cytovillin) (Villin-2) (p81)	EZR
FKBP1A	P62942	Peptidyl-prolyl cis-trans isomerase FKBP1A	FKBP1A
FKBP1B	P68106	Peptidyl-prolyl cis-trans isomerase FKBP1B	FKBP1B
FPs	P07332	Tyrosine-protein kinase Fes/Fps	FES FPS
S glycoprotein	P0DTC2	S glycoprotein	S2

GRP78	P11021	Endoplasmic reticulum chaperone BiP	GRP78
HR1	Q96GN5	Cell division cycle-associated 7-like protein	
HSP27	Q15424	Scaffold attachment factor B1, SAF-B, SAF-B1	SAFB
IFITM1	P13164	Interferon-induced transmembrane protein 1	IFITM1
IFITM3	Q01628	Interferon-induced transmembrane protein 3	IFITM3
IFN regulatory factor 3	Q14653	Interferon regulatory factor 3	IRF-3
IFN regulatory factor 9	Q00978	Interferon regulatory factor 9	IRF-9
IL-12	P29459, Q99665	Interleukin 12, subunit alpha	IL-12
IL-6	P05231	Interleukin-6	IL6
IL-8	P10145	Interleukin-8	CXCL8, IL8
interferon gamma	P17803	Interferon gamma, IFN-gamma	IFNG
interleukin 2	P60568	Interleukin-2	IL2
IRF-1	P10914	Interferon regulatory factor 1, IRF-1	IRF-1
IRF3	Q14653	Interferon regulatory factor 3, IRF-3	, IRF-3
LFP	Q92974	Rho guanine nucleotide exchange factor 2	ARHGEF2
Man9GlcNAc	Q9BVK2	Probable dolichyl pyrophosphate Glc1Man9GlcNAc2 alpha-1,3-glucosyltransferase	ALG8
Man9GlcNAc2	Q9Y672	Dolichyl pyrophosphate Man9GlcNAc2 alpha-1,3-glucosyltransferase	ALG6
MAVS	Q7Z434	Mitochondrial antiviral-signaling protein, MAVS	MAVS
MBL	P11226	Mannose-binding protein C, MBP-C	MBL2
MyD88	Q99836	Myeloid differentiation primary response protein MyD88	MYD88
NFAT	Q8NET5	NFAT activation molecule 1	NFAM1

orf9b	P59636	Protein 9b	9b
p53	P04637	Cellular tumor antigen p53	TP53
PARP1	P09874	Poly [ADP-ribose] polymerase 1	PARP1
PPIA	P62937	Peptidyl-prolyl cis-trans isomerase A	PPIA
PPIB	P23284	Peptidyl-prolyl cis-trans isomerase B	PPIB
PPIH	O43447	Peptidyl-prolyl cis-trans isomerase H, PPIase H	PPIH
PPIG	Q13427	Peptidyl-prolyl cis-trans isomerase G	PPIG
RCHY1	RCHY1	RING finger and CHY zinc finger domain-containing protein 1	RCHY1
RIG-I	O95786	Antiviral innate immune response receptor RIG-	DDX58
SMAD3	P84022	Mothers against decapentaplegic homolog 3	SMAD3
STAT1	P42224	Signal transducer and activator of transcription 1-alpha/beta	STAT1
STAT6	P42226	Signal transducer and activator of transcription 6	STAT6
TBK1	Q9UHD2	Serine/threonine-protein kinase TBK1	TBK1
TGF-β1	Q9H2G4	Testis-specific Y-encoded-like protein 2	TSPYL2
TLR7	Q9NYK1	Toll-like receptor 7	TLR7
TLR8	Q9NR97	Toll-like receptor 8	TLR8
TMPRSS2	O15393	Transmembrane protease serine 2	TMPRSS2
TNF	P01375	Tumor necrosis factor	TNF
TRAF3	Q13114	TNF receptor-associated factor 3	TRAF3
trypsin	P07477	Trypsin-1	PRSS1
vimentin	P08670	Vimentin	VIM
p42	P62333	26S proteasome regulatory subunit 10B	PSMC6
HLA-A2	P04439	HLA class I histocompatibility antigen	HLA-A

IL-1β	P27930	Interleukin-1 receptor type 2	IL1R2
Arf1	P84077	ADP-ribosylation factor 1	ARF1
ISG15	P05161	Ubiquitin-like protein ISG15	ISG15
aminopeptidase N	P15144	Aminopeptidase N	ANPEP
MHC class II	P20036	HLA class II histocompatibility antigen	HLA-DPA1

Table S4. The list of human proteins identified as having impact on SARS-CoV-2 and Dengue virus infections development.

Name_found	Unirot	Name	Species
Axl	P30530	Tyrosine-protein kinase receptor UFO	Homo sapiens
chaperone	Q16543	Hsp90 co-chaperone Cdc37	Homo sapiens
E3	Q9H6Y7	E3 ubiquitin-protein ligase RNF167	Homo sapiens
FPs	P07332	Tyrosine-protein kinase Fes/Fps	Homo sapiens
GRP78	P11021	Endoplasmic reticulum chaperone BiP	Homo sapiens
IFITM1	P13164	Interferon-induced transmembrane protein 1	Homo sapiens
IFITM3	Q01628	Interferon-induced transmembrane protein 3	Homo sapiens
IFN regulatory factor 3	Q14653	Interferon regulatory factor 3	Homo sapiens
IL-10	P22301	Interleukin-10	Homo sapiens
IL-6	P05231	Interleukin-6	Homo sapiens
IL-8	P10145	Interleukin-8	Homo sapiens
interferon regulatory factor 3	Q14653	Interferon regulatory factor 3	Homo sapiens
IRF3	Q14653	Interferon regulatory factor 3	Homo sapiens
M protein	A1A4Y4	Immunity-related GTPase family M protein	Homo sapiens
MAVS	Q7Z434	Mitochondrial antiviral-signaling protein, MAVS	Homo sapiens
MBL	P11226	Mannose-binding protein C	Homo sapiens
MMP	Q9H306	Matrix metalloproteinase-27	Homo sapiens
NF-kappa B	P19838	Nuclear factor NF-kappa-B	Homo sapiens
NPC1	O15118	NPC intracellular cholesterol transporter 1	Homo sapiens
NS1	Q9Y6Y0	Influenza virus NS1A-binding protein	Homo sapiens
SMAD7	O15105	Mothers against decapentaplegic homolog	Homo sapiens
STAT1	P422247	Signal transducer and activator of transcription 1-alpha	Homo sapiens
TBK1	Q9UHD2	Serine/threonine-protein kinase TBK1	Homo sapiens
TIM1	Q96D42	Hepatitis A virus cellular receptor 1	Homo sapiens
TIM3	Q8TDQ0	Hepatitis A virus cellular receptor 1	Homo sapiens
TIM4	Q96H15	T-cell immunoglobulin and mucin domain-containing protein 4	Homo sapiens
TLR3	O15455	Toll-like receptor 3	Homo sapiens
TNF	P01375	Tumor necrosis factor	Homo sapiens

Toll	Q15399	Toll-like receptor 1	Homo sapiens
VLPs	P62760	Visinin-like protein 1	Homo sapiens
